# The 15kDa Selenoprotein and Thioredoxin Reductase 1 Promote Colon Cancer by Different Pathways

**DOI:** 10.1371/journal.pone.0124487

**Published:** 2015-04-17

**Authors:** Petra A. Tsuji, Bradley A. Carlson, Min-Hyuk Yoo, Salvador Naranjo-Suarez, Xue-Ming Xu, Yiwen He, Esther Asaki, Harold E. Seifried, William C. Reinhold, Cindy D. Davis, Vadim N. Gladyshev, Dolph L. Hatfield

**Affiliations:** 1 Department of Biological Sciences, Towson University, Towson, Maryland, United States of America; 2 Molecular Biology of Selenium Section, Mouse Cancer Genetics Program, National Institutes of Health, Bethesda, Maryland, United States of America; 3 Center for Information Technology, National Institutes of Health, Bethesda, Maryland, United States of America; 4 Nutritional Science Research Group, National Institutes of Health, Rockville, Maryland, United States of America; 5 Genomics & Informatics Group, Laboratory of Molecular Pharmacology, National Institutes of Health, Bethesda, Maryland, United States of America; 6 Office of Dietary Supplements, National Institutes of Health, Rockville, Maryland, United States of America; 7 Brigham and Women’s Hospital, Harvard Medical School, Boston, Massachusetts, United States of America; University of South Alabama Mitchell Cancer Institute, UNITED STATES

## Abstract

Selenoproteins mediate much of the cancer-preventive properties of the essential nutrient selenium, but some of these proteins have been shown to also have cancer-promoting effects. We examined the contributions of the 15kDa selenoprotein (Sep15) and thioredoxin reductase 1 (TR1) to cancer development. Targeted down-regulation of either gene inhibited anchorage-dependent and anchorage-independent growth and formation of experimental metastases of mouse colon carcinoma CT26 cells. Surprisingly, combined deficiency of Sep15 and TR1 reversed the anti-cancer effects observed with down-regulation of each single gene. We found that inflammation-related genes regulated by Stat-1, especially interferon-γ-regulated guanylate-binding proteins, were highly elevated in Sep15-deficient, but not in TR1-deficient cells. Interestingly, components of the Wnt/β-catenin signaling pathway were up-regulated in cells lacking both TR1 and Sep15. These results suggest that Sep15 and TR1 participate in interfering regulatory pathways in colon cancer cells. Considering the variable expression levels of Sep15 and TR1 found within the human population, our results provide insights into new roles of selenoproteins in cancer.

## Introduction

Colon cancer remains the second leading cause of cancer-related deaths [1]. Supplemental dietary selenium has been reported to reduce the incidence and mortality from colon cancer in humans [2] and animals with suboptimal selenium levels [3]. These cancer-preventive properties are primarily mediated through selenoproteins [4], suggesting an underlying genetic susceptibility to colon cancer. Three selenoproteins have been implicated in both prevention and promotion of cancer: the 15kDa selenoprotein (Sep15) [5–7], thioredoxin reductase 1 (Txnrd1, TR1) [8], and glutathione peroxidase 2 (GPx2) [9]. Furthermore, studies of single nucleotide polymorphisms revealed an association of both TR1 and selenoprotein P (SEPP1) with advanced colorectal adenomas in humans [10]. We previously examined the dual personalities of the first two selenoproteins [7,8,11,12], and here, we have further investigated their interactions in their regulation of colon cancer. Sep15 and TR1 belong to the thiol-oxidoreductase group of selenoproteins [13]. TR1 is a major redox-regulator in mammalian cells and is also involved in cell proliferation, angiogenesis, transcription, and DNA repair [14,15]. The physiological function of Sep15 remains poorly understood, but it may be involved in rearrangement of disulfide bonds or serve as a reductase for incorrectly formed disulfide bonds in misfolded glycoproteins bound to UDP-glucose:glycoprotein-glucosyl-transferase (UGGT) [16].

We previously demonstrated that loss of TR1 reversed the malignant properties of LLC1 mouse lung cancer cells [17]. Similarly, loss of Sep15 reversed the cancer phenotype of murine [18] and human colon cancer cells [19]. Because both proteins are selenoproteins, it is possible that they have additive or synergistic effects. Sep15 is differentially expressed in some human cancers [20,21], and TR1 is up-regulated in many cancers [14]. Herein, we examined the relationship between Sep15 and TR1 in mouse CT26 colon adenocarcinoma cells with regard to the roles of these selenoproteins in colon tumorigenesis. Interestingly, removal of these two selenoproteins appeared to achieve the reversal of cancer phenotypes through very different pathways, and, unexpectedly, a combined lack of Sep15 and TR1 was compensated by up-regulation of components in the Wnt/β-catenin signaling pathway.

## Materials and Methods

### Accession Codes

Microarray data are accessible through the Gene Expression Omnibus database (http://www.ncbi.nlm.nih.gov/geo; accession # GSE55488).

### Materials

Murine CT26 colon cancer cells were purchased from ATCC (Manassas, VA), and only low passage numbers were used. The pSilencer2.0 U6-Hygro-vector was from Ambion (ThermoFisher Scientific, Rockford, IL), fetal bovine serum, phosphate-buffered saline (PBS), RPMI 1640, hygromycin B, NuPAGE 4–12% polyacrylamide gels, SeeBlue Plus2 protein markers, lithium dodecylsulfate (LDS) sample buffer, Lipofectamine2000, polyvinylidene difluoride (PVDF) membranes, and TRIzol reagent from Invitrogen (Carlsbad, CA), and 5,5′-dithiobis(2-nitrobenzoic acid) and aurothioglucose from Sigma-Aldrich (St. Louis, MO). Antibodies against Sep15 and TR1 were generated in rabbits by Epitomics/Abcam (Cambridge, MA) using a synthetic peptide (Sep15) or recombinant protein (TR1) as antigens, and were validated in our lab for this company. Rabbit-anti-Alpha fetoprotein (Afp) antibody and horseradish peroxidase–conjugated secondary antibody against goat were obtained from Abcam (Cambridge, MA), and mouse anti-phospho-β-catenin primary antibody from Cell Signaling (Danvers, MA). Antibodies against β-actin, α-tubulin, guanylate binding-protein-1 (Gbp-1), Stat1 (p84/p91), and interferon-induced protein 44 (Ifi44) were from Santa Cruz Biotechnology (Dallas, TX). Horseradish peroxidase–conjugated secondary antibody against rabbit was from Sigma-Aldrich (St. Louis, MO). SuperSignal West Dura substrate and horseradish peroxidase–conjugated secondary antibody against mouse were from Pierce (ThermoFisher Scientific, Rockford, IL), and iScript cDNA Synthesis kit and SYBRGreen Supermix from Bio-Rad Laboratories (Hercules, CA). Real-time RT-PCR primers were from Sigma-Genosys (St. Louis, MO), noble agar from Becton Dickinson (Franklin Lakes, NJ), and black India ink from Winsor & Newton. Other reagents were of the highest commercially-available quality.

### Targeted down-regulation of Sep15, TR1, and TR1/Sep15

The pU6-m3 vector used for generating short hairpin RNA (shRNA) targets, and stable transfection of shSep15 and shTR1 CT26 colon cancer cells were constructed as described [17,18,22], and only low passage numbers were used for experiments. Sep15- and TR1-deficient and the corresponding CT26 parental cells were transfected with identical vectors (see [15,17] for details) except for the shRNAs. We investigated two shRNA oligonucleotide sequences, and several independent cell clones of these sequences resulted in very similar decreases in both mRNA and protein levels. The two shRNA oligonucleotide sequences for Sep15 and TR1 have been shown previously to result in similar reversal of cancer phenotype [17,18]. The double-knockdown cells incorporated both of the validated constructs. Cells with double-knockdown of Sep15 and TR1 together were prepared as described [22].

### Cell culture and cell growth assays

Mouse colon cancer CT26 cells were cultured in complete growth medium (RPMI1640 supplemented with 10% fetal bovine serum and 1% sodium pyruvate) in a humidified atmosphere (5% CO_2_, 37°C). Cells were stably transfected with shSep15, shTR1, a combination of the shTR1 and shSep15 constructs, or the empty pU6-m3 vector (control) using Lipofectamine2000 by selecting cells in the presence of 500 μg/mL hygromycin B. Cell growth was monitored by seeding 1×10^5^ cells/well in six-well plates in complete growth medium and counting cells for four days.

### RNA analysis

Total RNA was extracted from cells with TRIzol. cDNA was synthesized with iScript and 1.5 μg of total RNA according to the manufacturer’s instructions. For quantitative real-time RT-PCR (qPCR), 1.5 μL cDNA were added to 20-μL reactions with the DNA Engine Opticon2 Real-Time PCR Detection System (Bio-Rad Laboratories). The primers for qPCR are shown in S1 Table. mRNA levels of selenoproteins were calculated relative to the expression of glyceraldehyde-3-phosphate dehydrogenase (*Gapdh*), used as an internal control.

### Western blotting analyses

Protein samples extracted from the four stably-transfected CT26 cell lines were electrophoresed on NuPAGE 4–12% polyacrylamide gels followed by transferring to PVDF membranes. The membranes were incubated with primary antibodies (1:200 to 1:2,000) overnight at 4°C. Horseradish peroxidase–conjugated secondary antibodies (1:10,000) were applied, the membranes incubated in chemiluminescent substrate, and exposed to X-ray film.

### Thioredoxin reductase (TR) activity

TR activity was determined spectrophotometrically [23], based on the modified method of Holmgren and Björnstedt [24]. Briefly, TR activity was determined as the difference between total TR activity and time-dependent increase in absorbance at 412 nm in the presence of the TR inhibitor, aurothioglucose. A unit of activity was defined as 1.0 μmol 5-thio-2-nitrobenzoic acid formed/min/mg protein. Protein concentrations were measured with bicinchoninic acid.

### Soft agar assay

Anchorage-independent growth was examined by soft agar assays [17,18]. Unlike most cells with a normal phenotype, many cancer cells are able to grow unanchored in soft agar. Approximately 3,000 cells of each stably-transfected CT26 cell line were suspended in 3 mL of 0.35% noble agar in complete RPMI 1640 and spread onto 60-mm dishes masked with a basal layer of 3 mL 0.7% noble agar in media. Cells were incubated at 37°C for three weeks, and complete growth medium was applied to the dishes every three days. Colonies were visualized by staining overnight with *p*-iodonitrotetrazolium violet, scanned and counted.

### Cell cycle analysis

CT26 cells were grown in complete medium to 50% confluence, washed twice with sterile PBS, and maintained in serum-free medium for 48 h to induce G0–G1 cell cycle synchronization. Cells were then incubated with complete growth medium for 0, 6, 24 or 48 h, washed twice with PBS, trypsinized, and suspended in PBS (1×10^7^ to 2×10^7^ cells/mL) on ice. Ice-cold 70% ethanol was added gradually, and cells were fixed overnight. Cells were centrifuged, resuspended in RNase (100 units), and incubated at 37°C for 20 min. The suspension was stained with propidium iodide in the dark at 4°C overnight, filtered through a 50-μm mesh, and acquired with a FACSCalibur (Becton Dickinson, San Jose, CA). The percentage of cells in each phase of the cell cycle was analyzed by ModFitLT version 3.0 (Verity, Topsham, ME).

### Animal studies

This study was carried out in strict accordance with the recommendations in the Guide for the Care and Use of Laboratory Animals of the National Institutes of Health. The protocol was approved by the NCI-Bethesda Animal Care and Use Committee (permit number for this study is LCP-005). Formation of experimental lung metastases upon *i*.*v*. injection of plasmid-transfected control, shSep15, shTR1, or shTR1/shSep15 cells was investigated in BALB/c mice (Jackson Laboratories), which have the same genetic background as CT26 adenocarcinoma cells. Animals were given free access to water and were monitored closely for clinical signs of poor health throughout the study. Six-week-old male BALB/c mice were maintained on a Torula yeast-based diet supplemented with adequate selenium (0.1 μg selenium/g diet) as sodium selenite (Harlan-Teklad, Indianapolis, IN). After three weeks, 5×10^5^ CT26 cells in 200 μL PBS (*n* = 10 each for plasmid-transfected controls, shSep15, shTR1, or shTR1/shSep15 cells) were injected into the tail vein. Animals were sacrificed, and lungs were examined 12 days after injection. Three milliliters of 15% India ink/PBS solution were injected into the lungs through the trachea. Lung tissues were excised and “bleached” with Fekete's solution (60% ethanol, 3.2% formaldehyde, and 0.75 mol/L acetic acid) for 48 h. Pulmonary lesions on the surface of each lobe were counted with a dissecting microscope. Lungs with >250 lesions were assigned a value of 250, as it is inherently difficult to reliably count more than 250 lesions/lung [18].

### Microarray analysis

Total mRNA was isolated from plasmid-transfected control, shSep15, shTR1 and shTR1/shSep15 CT26 cells. Microarray analysis was performed on Affymetrix Mouse 430_2.0A gene chips. Four arrays were analyzed from four different mRNA samples per construct. The Robust Multi-Array Analysis algorithm was used for background and noise correction in the conversion of probe level to gene expression data. Controls and cells with targeted down-regulation of Sep15, TR1, or TR1/Sep15 were compared by ANOVA. Genes that were significantly different from plasmid-transfected control cells (*P<*0.05) were subjected to functional gene analysis by Ingenuity Pathway Analysis (IPA; version 7.5), which detects networks of gene expression changes according to functional biological processes rather than focusing solely on the more dramatic changes in expression of a few selected genes. Previously published, and publicly available, microarray data from a study using Sep15 knockout mice were accessed for comparison purposes [7].

### Statistical analyses

Data presented as the mean ±SE were analyzed by one-way ANOVA followed by Tukey's multiple-comparison *post-hoc* test with GraphPad Prism (version 5, La Jolla, CA). The level of significance was set at α = 0.05, and statistically significant changes were indicated in figures as follows: *P<0.05, **P<0.01, ***P<0.001, compared to control. CellMiner’s NCI-60 Analysis Tool, which integrates molecular datasets of 60 human cancer cell lines routinely used for pharmacological screening and comparative molecular analyses, was used to identify genes significantly correlated with Sep15 expression (http://discover.nci.nih.gov/cellminer, database version 1.4) based on Pearson's correlation coefficient with a population size of 60, r = 0.254, and *P<*0.05 without multiple comparisons correction. Gene transcript expression patterns were also compared. CellMiner bases transcript intensity levels on measurements from five microarrays (Affymetrix HG-U95, HG-U133, HG-U133 plus 2.0, and HuEx 1.0, and the Agilent Whole Human Genome Microarray) as described [25].

## Results

### Individual and concurrent deficiency in TR1 and Sep15

**Fig 1:**
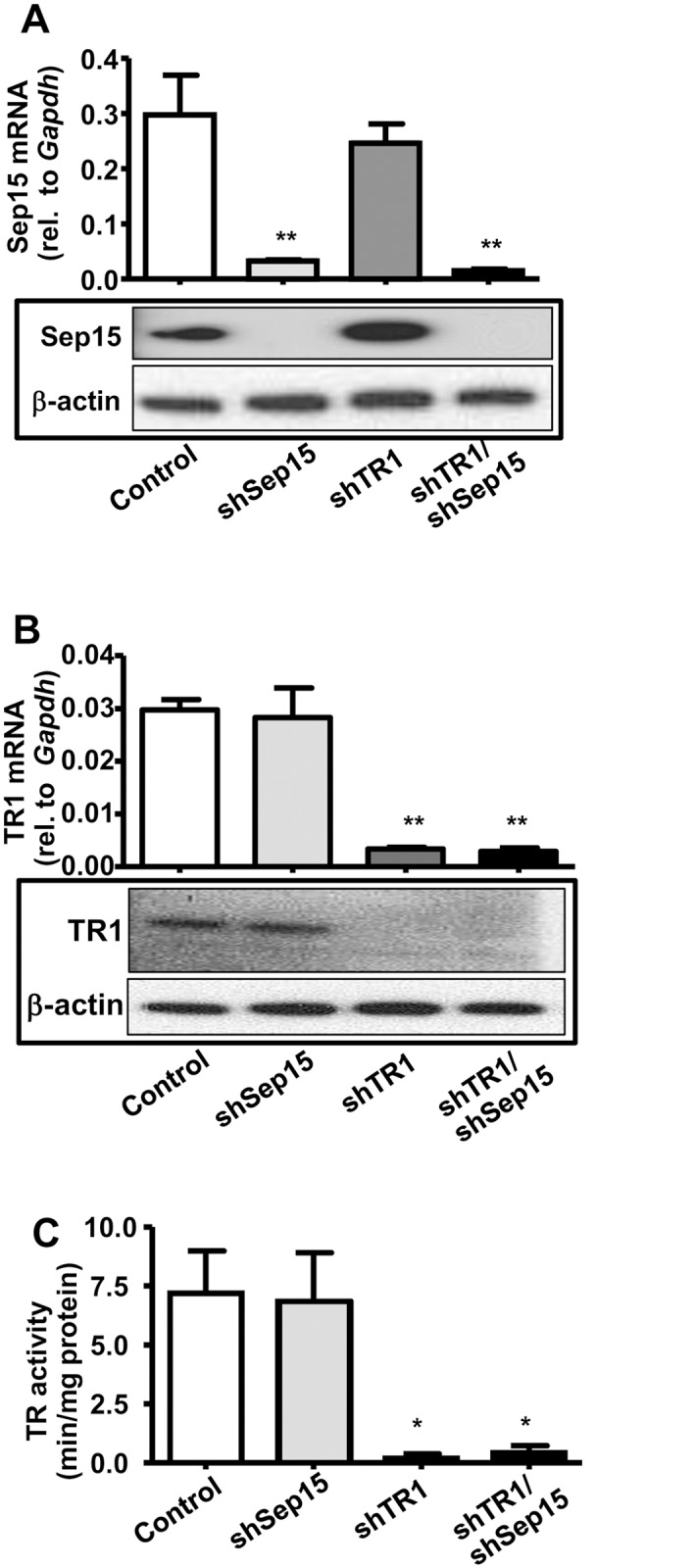
Expression of Sep15 and TR1 in CT26 cells. Cells were stably transfected with the pU6-m3 control, shSep15, shTR1 or shTR1/shSep15 (as indicated). **(A)** Expression of Sep15 by real-time RT-PCR (upper panel) and Western blotting (lower panel). **(B)** Expression of TR1 by real-time RT-PCR (upper panel) and Western blotting (lower panel). **(C)** Thioredoxin reductase activity. Columns, mean (*n* = 3–6); bars, SE; (*P<0.05, **P<0.01, compared to control).

Mouse colon cancer CT26 cells were stably transfected with shSep15, shTR1, shTR1/shSep15, or the corresponding pU6-m3-(empty vector)-control constructs. We used two shRNA oligonucleotides, and several independent cell clones were chosen for analysis to exclude off-target effects due to the constructs or the transfection process, as published [17,18]. qPCR and Western blotting showed that Sep15 mRNA (Fig 1A, upper panel) and protein levels (Fig 1A, lower panel) were efficiently (>90%) decreased in cells transfected with shSep15 or shTR1/shSep15. qPCR, Western blotting, and catalytic activity assays showed that TR1 mRNA (Fig 1B, upper panel) and protein expression (Fig 1B, lower panel), as well as catalytic activity (Fig 1C) were decreased by >90% in shTR1 and shTR1/shSep15 cells. The mRNA expression of glutathione peroxidases 1 (GPx1) and 2 (GPx2) were not significantly changed, and selenoprotein M (SelM) was only modestly increased in shSep15 (*P*<0.05) and shTR1/Sep15 cells (*P*>0.05), as expected (S1 Fig).

### shSep15 or shTR1 inhibit cell proliferation and development of experimental metastases

**Fig 2:**
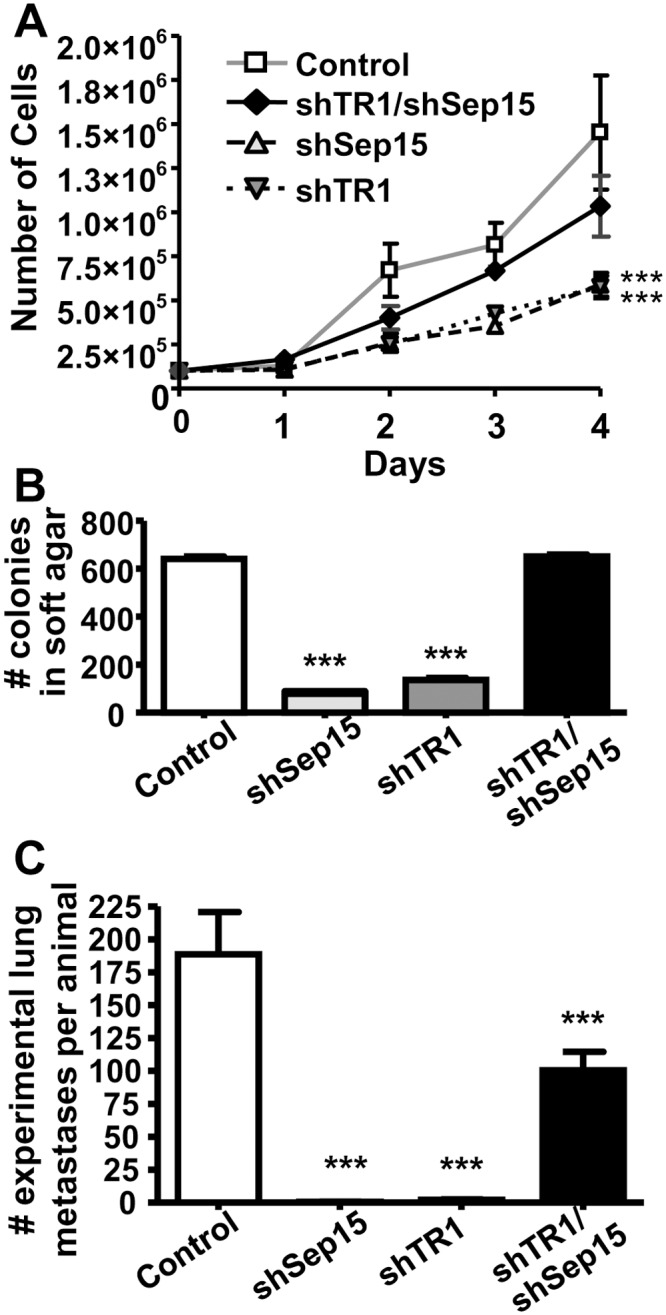
Anchorage-dependent, -independent growth and formation of lung metastases. (**A**) Growth rates of shSep15, shTR1 and shTR1/Sep15 cells compared to controls (*n* = 6). (**B**) Anchorage-independent growth in soft agar of shSep15, shTR1 and shTR1/Sep15 cells compared to controls (*n* = 4–8). (**C**) Formation of experimental lung metastases after i.v. injection of 5×10^5^ cells (control, shSep15, shTR1, or shTR1/shSep15) into BALB/c mice (*n* = 10/construct). (***P<0.001, compared to control).

Anchorage-dependent proliferation of both shSep15 and shTR1 cells was significantly reduced (*P<*0.001) (Fig 2A). In each case, there were 60% fewer cells on day 4 compared to controls. Surprisingly, the growth pattern of shTR1/shSep15 cells was similar to control cells. shSep15 and shTR1-transfected CT26 cells formed significantly (*P*<0.001) fewer colonies than control cells in anchorage-independent growth assays (Fig 2B). In contrast, TR1/Sep15 knockdown did not affect colony growth in soft agar.

Control cells formed a large number (188.6±32.0) of experimental lung metastases in BALB/c mice (Fig 2C), as expected. As also previously shown [18], targeted reduction of Sep15 significantly (*P<*0.001) inhibited formation of such pulmonary metastases (0.5±0.2). Similarly, shTR1 cells formed fewer experimental metastases (2±0.4) compared to controls (*P<*0.001). Mice injected with shTR1/shSep15 cells developed fewer experimental metastases than controls (100.4±14.1; *P*<0.001), but over 50-times more (*P<*0.01) compared to those injected with single knockdown cells.

### shTR1/shSep15 reverses the cell cycle phenotypes observed in single knockdown cells

**Fig 3:**
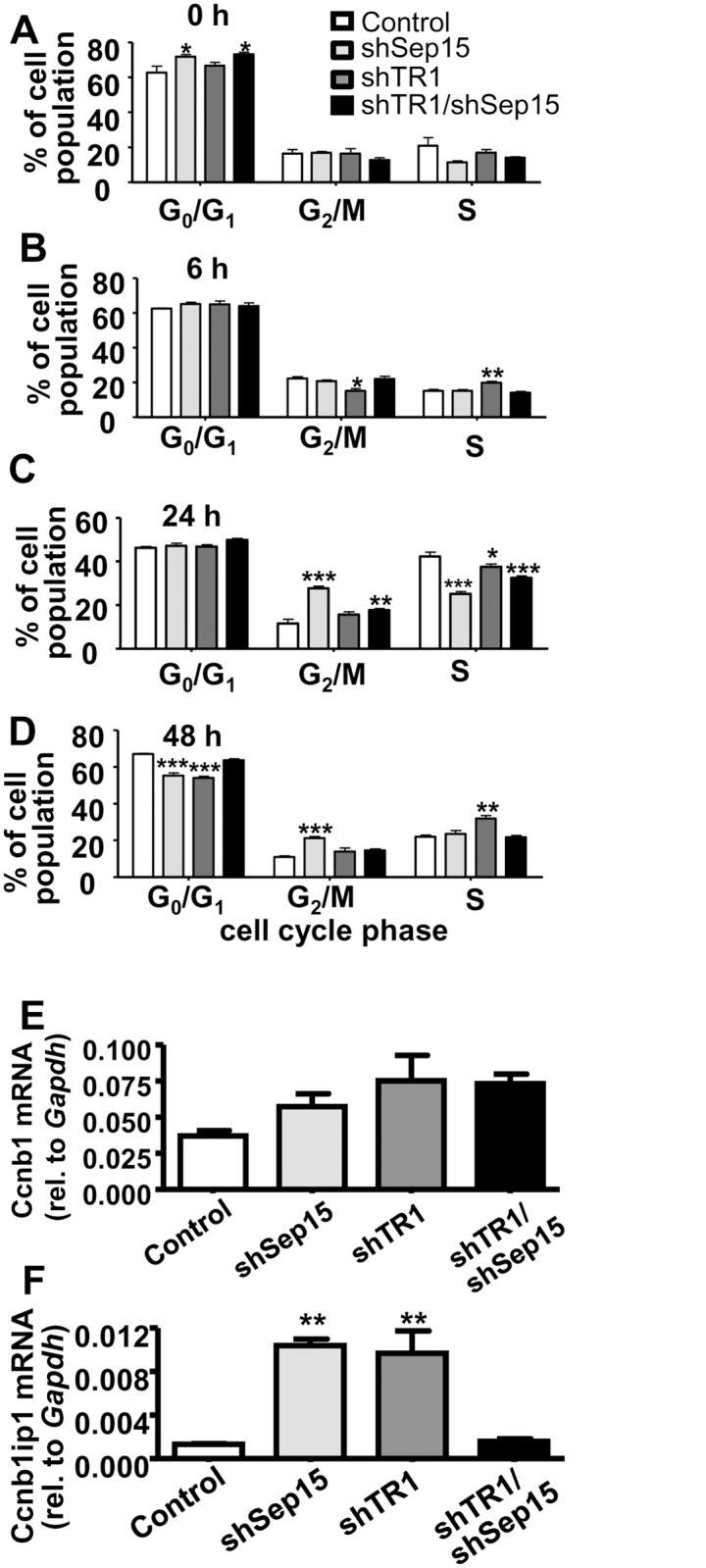
Cell cycle analysis. Percent of cells in each cell cycle phase as determined by FACS analysis at (**A**) 0 h; (**B**) 6 h; (**C**) 24 h; and (**D**) 48 h after release from cell synchronization. mRNA expression of (**E**) CyclinB1 (*Ccnb1*), and (**F**) CyclinB1 Interacting-Protein-1 (*Ccnb1ip1*), as determined by real-time RT-PCR. Columns, mean (*n* = 3–9); bars, SE; (*P<0.05, **P<0.01, ***P<0.001).

The most striking cell cycle changes were observed in shSep15 cells (Fig 3A–3D), and agree with our previously published observations, that the most significant differences between shSep15 and control cells appeared around 24 h past release from serum starvation [18]. shSep15 cells had a substantially higher percentage of their population in the G2/M-phase (27.8±0.8%) than control cells (11.5±2.1%; *P<*0.001), and a smaller percentage in S-phase (25.1±0.9% versus 42.3±1.9%), similar to earlier observations [18]. However, shTR1 and shTR1/shSep15 cells had fewer cells in G2/M-phase (15.6±1.2% and 17.7±0.7%, respectively) compared to shSep15 cells (*P<*0.001). shTR1 cells, as observed in fibroblast-derived DT cells previously [15], manifested defective progression in S-phase, visible as an increased percentage of the cell population at 6 and 48 h. CyclinB1 (Ccnb1) mRNA levels did not significantly differ (Fig 3E), but the mRNA levels of CyclinB1-interacting-protein-1 (Ccnbip1) were strongly elevated in shTR1 and shSep15 cells (*P<*0.01), but not in shTR1/shSep15 cells, compared to controls, respectively (Fig 3F).

### Gene expression analyses identify interferon-γ- and Wnt/β-catenin-regulated pathways in shSep15 and shTR1/shSep15 cells, respectively

To elucidate the signaling pathways affected by Sep15- and TR1-deficiency in colon cancer cells, and reversal of these effects in double knockdown cells, global gene expression was analyzed using microarrays (N = 4/construct). The twenty most substantially altered gene signals are listed in S2 Table. Selected genes were examined by qPCR relative to the expression of an internal control, *Gapdh*, to validate microarray results.

**Fig 4:**
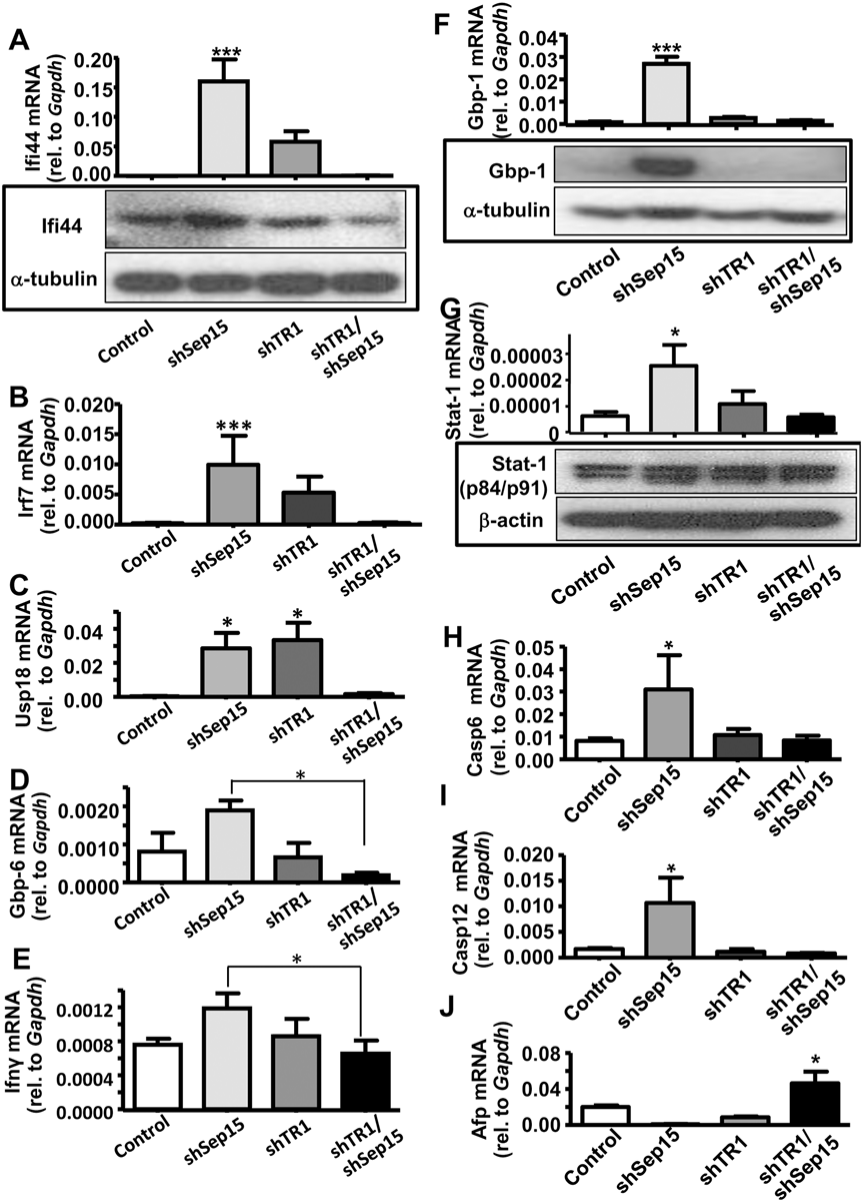
Validation of gene expression for genes significantly changed in shSep15 cells. Expression of **(A)** Ifi44 mRNA (upper panel) and protein (lower panel); **(B)** Irf-7 mRNA; **(C)** Usp18 mRNA; (**D**) Gbp-6 mRNA; **(E)** Ifnγ mRNA; (**F**) Gbp-1 mRNA (upper panel) and protein (lower panel); (**G**) Stat-1 mRNA (upper panel) and protein (lower panel); (**H**), Casp6 mRNA; (**I**) Casp12 mRNA; and (**J**) Afp mRNA. Expression of mRNA in control, shSep15, shTR1, and shTR1/Sep15 cells was determined by real-time RT-PCR, and expressed relative to *Gapdh*. Columns, mean (*n* = 3–6); bars, SE; (*P<0.05, **P<0.01, ***P<0.001). Protein expression was determined by Western blotting, and expressed relative to β-actin or α-tubulin, as indicated.

The most strongly up-regulated gene signals in shSep15 cells were interferon-induced-protein-44 (*Ifi44*), ubiquitin-specific-peptidase-18 (*Usp18*), immunity-related-GTPase family-M-member-2 (*Irgm2*), interferon-regulatory-factor-7 (*Irf7*), interferon-γ-induced-GTPase *(Igtp*), very large interferon-inducible-GTPase *(Gvin1*), and guanylate-binding-proteins *Gbp-1*, *-2* and *-6*. Significantly increased mRNA expression of *Ifi44* (*P*<0.001, Fig 4A, upper panel), *Irf7* (*P*<0.001, Fig 4B), and *Usp18* (*P*<0.05, Fig 4C) in shSep15 cells compared to controls were validated with qPCR. Protein expression levels of Ifi44 (Fig 4A, lower panel), were slightly increased in shSep15 cells. The mRNA levels of *Gbp-6* (*P<*0.05; Fig 4D) and interferon-γ (Ifnγ) (*P<*0.05; Fig 4E) were significantly higher in shSep15 compared to shTR1/shSep15 cells. The levels of Gbp-1 mRNA were previously reported to be highly up-regulated in Sep15 knockout mice [7]. Consistent with these observations, we found that Gbp-1 mRNA (Fig 4F, upper panel) and protein levels (Fig 4F, lower panel) were significantly increased exclusively in shSep15 cells. Ingenuity Pathway Analyses (IPA) indicated that inflammation-related genes up-regulated in shSep15 cells shared a common node in *Stat-1* (S3A Fig), and included *Gbp-2* (31.1-fold), *Gbp-6* (30.1-fold), *Nmi* (2.8-fold), *Irgm2* (20.7-fold), and *Gbp-1* (14.3-fold; included in ‘Gbp-2*’). Other significantly up-regulated *Stat-1*-associated genes included *Usp18* (65.1-fold), *Irf7* (9.9-fold) and *Irf9* (3.6-fold). *Stat-1* mRNA was quantified with qPCR and found to be significantly increased only in shSep15 cells compared to controls (*P<*0.01, Fig 4G, upper panel), but protein levels of unphosphorylated Stat-1 remained unchanged (Fig 4G, lower panel). Stat-1 is thought to promote activation of different caspases, and the mRNA levels of caspase 6 (Fig 4H) and caspase 12 (Fig 4I) were significantly increased only in shSep15 cells (*P*<0.05). Supporting this pattern of involvement of interferon-γ and Sep15 are additional comparisons with our previously published microarray data [7], which showed a modest but significantly (*P*<0.05) increased mRNA expression of interferon-γ-regulated Stat-1, Stat-5A, Irf-2, and Irf-3, in Sep15 knockout mice (N = 4) compared to litter mate controls (N = 4). The gene most significantly down-regulated in shSep15 cells was alpha-fetoprotein (*Afp*). Interestingly, mRNA levels of *Afp* were increased 1.5-fold in shTR1/shSep15 cells as determined by qPCR (Fig 4J), but protein levels remained unchanged (S2A Fig).

**Fig 5:**
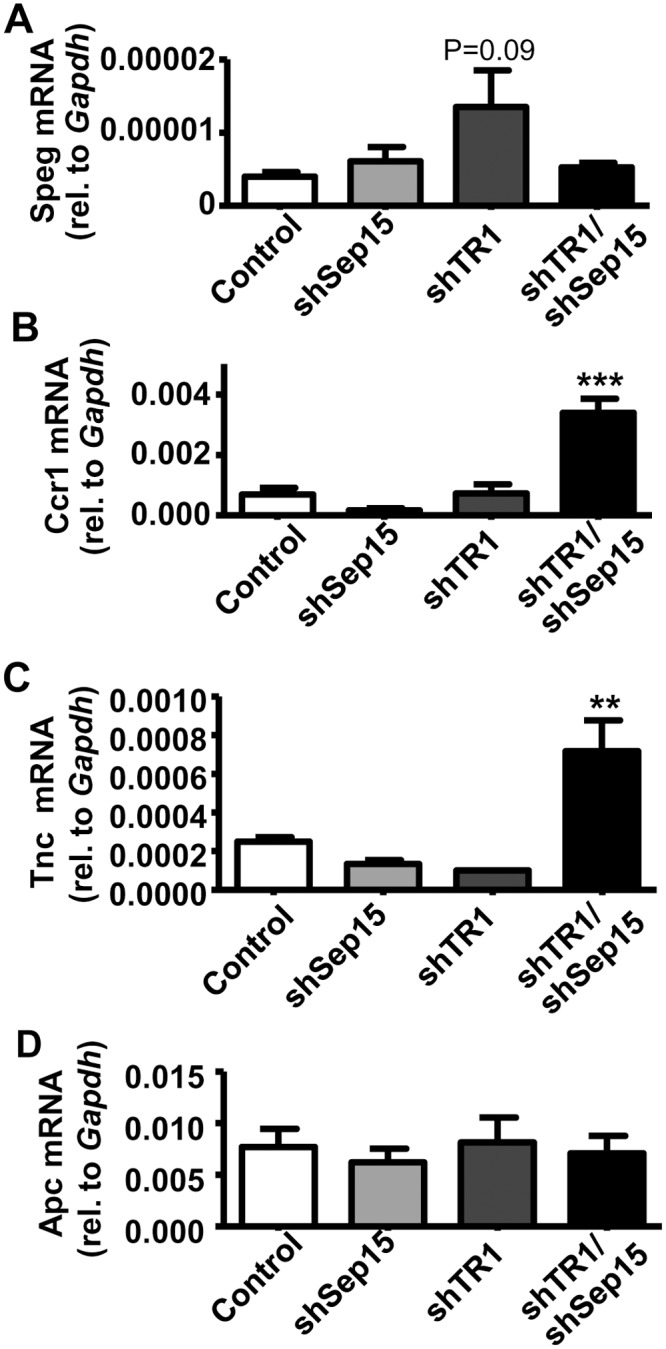
Possible connection to Wnt/β-catenin signaling pathway in shTR1/Sep15 cells. mRNA levels of **(A)** Speg; **(B)** Ccr1; and **(C)** Tnc; and (**D**) Apc in control, shSep15, shTR1, and shTR1/Sep15 cells, as determined by real-time RT-PCR, and expressed relative to *Gapdh*. Columns, mean (*n* = 3–6); bars, SE; (**P<0.01, ***P<0.001).

In the microarray analyses, genes with significantly altered expression in shTR1 cells compared to controls (S2 Table) included chemokine receptor-type-1 (*Ccr1*, 9.0-fold), which was also increased in shTR1/shSep15 cells (5.1-fold), and the striated preferentially-expressed-gene (*Speg* 5.8-fold), which is known to inhibit cell proliferation [26]. Subsequent qPCR analyses showed an about two-fold increase in Speg mRNA expression in shTR1 cells compared to controls (*P* = 0.09, Fig 5A), and only significant increases in Ccr1 mRNA expression in shTR1/Sep15 cells compared to controls (*P*<0.001, Fig 5B). The mRNA levels of the cell cycle regulator *Ccnb1ip1*, which was also previously reported as dramatically increased in shSep15 CT26 cells [18], were also increased in shTR1 cells (*P*<0.01, Fig 3F). Usp18 mRNA expression, which microarray analyses identified as 16-fold higher (*P* = 0.09) in shTR1 cells, were also significantly increased (*P*<0.05) as validated by qPCR (Fig 4C). Significant gene changes in shTR1 cells included those involved in DNA repair, redox regulation, ATP-binding cassette transport, ubiquitination and cancer-related pathways.

Combined down-regulation of Sep15 and TR1 resulted in rather unexpected changes. Microarray analyses revealed that the most up-regulated genes that were exclusively changed in the shTR1/shSep15 cells (S2 Table) compared to controls were tenascin (*Tnc*, 9.0-fold) and the mitogen-regulated prolactin-family-2 subfamily-c-member-2 (*Prl2c2*, 7.5-fold). Significant up-regulation of tenascin mRNA (*P*<0.01) was subsequently verified by qPCR (Fig 5C). IPA was performed for the 984 gene signals that were significantly different between shTR1/shSep15 and controls, but were not different in either of the single-knockdown cells versus controls. Two networks involved in *Apc/Wnt/β-catenin* pathways were identified (S3B and S3C Fig), which may compensate for combined loss of Sep15 and TR1. Interestingly, tenascin was indirectly networked with *Wnt* (S3C Fig). Expression levels of the adenomatous polyposis coli gene (*APC*, S3B Fig) and the negative regulator of the wingless-type (*Wnt*) genes, *Axin1*, were significantly but only modestly reduced (1.4-fold, respectively) in microarray analyses. Subsequent qPCR did not detect any differences in *Apc* mRNA expression among constructs (Fig 5D). Other *Wnt*-type genes (*e*.*g*., *Wnt10a*) and β-catenin-interacting protein-1 (*Ctnnbip1*) were significantly up-regulated (1.9-fold and 1.6-fold, respectively) in microarray analyses. However, protein levels of phosphorylated β-catenin itself, as assayed through Western blotting, appeared unchanged (S2B Fig).

### Sep15 expression is negatively correlated with Stat-1/Stat-2 and Usp18 in NCI-60 cell lines

**Fig 6:**
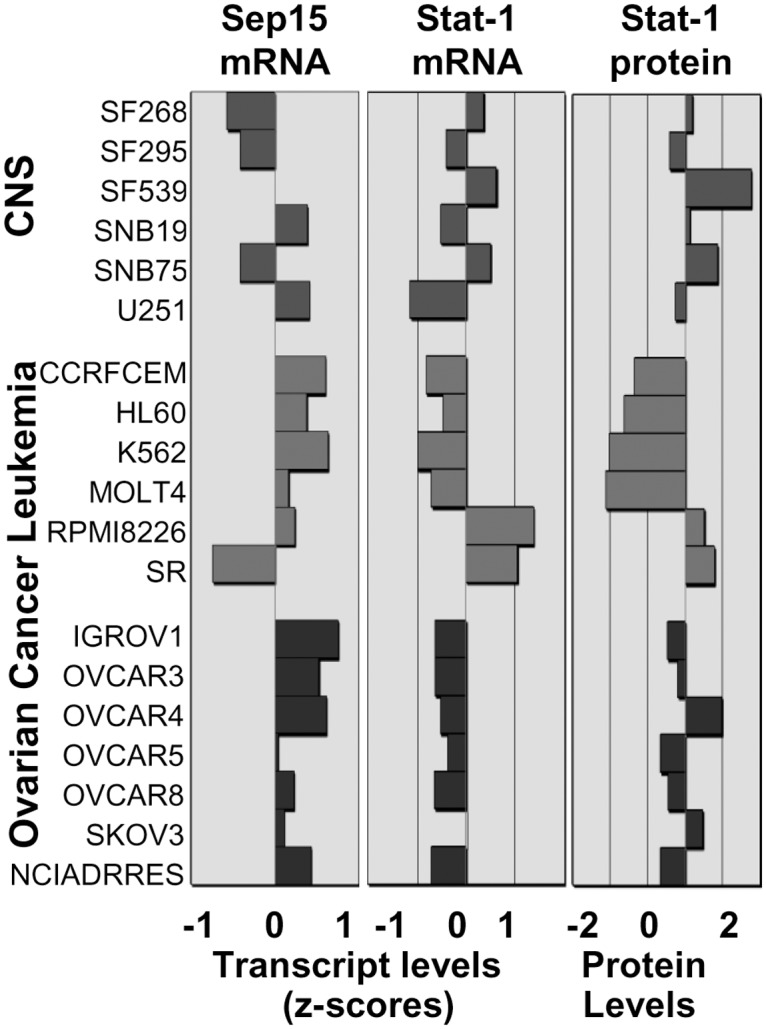
Signatures of selected NCI-60 cell lines for Sep15 and Stat-1. Opposing directions of transcript intensity scores (z-scores) for Sep15 and Stat-1 mRNAs were highly significant (r = -0.646, *P<*0.01) for human central nervous system (CNS), leukemia and ovarian cancer cell lines. Mean centered average protein activity patterns for Stat-1 (Stat-1_20 antibody) significantly correlated (r = 0.661, *P<*0.01) with Stat-1 mRNA expression.

In the NCI-60 cell lines, CellMiner negatively correlated (*P<*0.05) mRNA expression of Sep15, but not TR1, with Stat-2 (Pearson’s coefficient: -0.29) and with Usp18 (Pearson’s coefficient: −0.43), the second highest up-regulated gene signal in cells lacking Sep15. Comparison of cell line signatures revealed that the opposing directions of mRNA transcript intensity scores (z-scores) for Sep15 and Stat-1 (Fig 6) were particularly significant (r = −0.65, *P<*0.01) for human cancer cell lines derived from central nervous system, leukemia and ovarian cancers. Stat-1 protein levels in NCI-60 cell lines supported the negative correlation with Sep15 mRNA expression (Fig 6).

## Discussion

Our objective has been to understand the roles of the selenoproteins Sep15 and TR1 in cancer initiation and development [8,11,17–19,27]. The current study exposed unexpected complexities and interactions between these two selenoproteins in their regulation of colon cancer. In light of the benefits of increased selenium intake found in colon cancer prevention [28,29], and the controversial results in the recent human clinical trial SELECT [30], wherein no benefits were found, our results are particularly relevant as dietary selenium’s cancer-preventive properties are primarily mediated through selenoproteins [31].

The selenoproteins Sep15 and TR1 have important roles in cellular redox-homeostasis. The differences between these two systems are clear from *in vivo* studies. A complete lack of cytosolic TR1 is embryonic lethal [12], whereas a systemic Sep15 deficiency does not result in an obvious phenotype in mice other than formation of cataracts [32]. Targeted down-regulation of these two genes provides effective means to investigate their function, regulation and interactions. Our results in mouse colon cancer cells demonstrate that differences in cell cycle regulation may have partially contributed to reversals of the observed cancer phenotypes. However, the underlying mechanism eliciting this phenotype appears to differ between cells lacking Sep15 versus those lacking TR1. Sep15 deficiency was previously found to inhibit cell proliferation in mouse [18] and human colon cancer cells [19], but not in mouse lung cancer cells [18]. In contrast, lack of TR1 significantly reduced the growth of mouse lung cancer cells [17]. Considering the similar effects of Sep15 and TR1 on the cancer phenotype, lack of both selenoproteins was anticipated to yield additive or synergistic responses. Surprisingly, this combined down-regulation failed to achieve effects observed with single knockdown. To investigate the molecular targets that were affected by single versus double knockdown, global gene expression changes were examined.

The gene most significantly down-regulated in shSep15 cells, alpha-fetoprotein (*Afp*), is a widely-used tumor marker for hepatocarcinoma known to interact with *Notch* [33], and is increased during tumorigenesis [34]. Thus, suppression of *Afp* in shSep15 cells might contribute to the observed anti-cancer effects. More importantly, loss of Sep15 alone in colon cancer cells appears to induce interferon-γ-regulated gene expression, whereas a lack of both Sep15 and TR1 resulted in up-regulation of components of the Wnt/β-catenin pathway, presumably allowing these cells to compensate for these oxidoreductases. Pathway analysis of the genes that were significantly different between shSep15 cells and controls indicated that many up-regulated interferon-regulated genes (*e*.*g*., *Usp18*, *Gbp-1*, *-2*, *-6*, *Nmi*, *Irf7*, *Irf9*, *Irgm2*) shared *Stat-1* as a common node. Stat-1 is a central mediator of interferons and exerts anti-tumor effects. Its activation induces genes typical of a type-I immune response. Thus, Stat-1 activation through lack of Sep15 may lead to classical activation of macrophages, resulting in anti-proliferative and anti-angiogenic activities. This may also help further explain our previous *in vivo* results, whereas Sep15 knockout mice appeared to be resistant to the formation of chemically-induced aberrant crypt foci [7]. Studies suggest that Stat-1 promotes activation of different caspase family members [35]. Here, our microarray and qPCR results indicated a greater than two-fold up-regulation of caspase-6 mRNA in shSep15 cells (Fig 4H), which may be an important apoptotic signaling event in colon cancer cells [36,37]. Similarly, mRNA levels of caspase-12, which is known to process and activate inflammatory cytokines, were only significantly up-regulated in shSep15 cells (Fig 4I). The strong, statistically significant negative correlations between Sep15 and Stat-2, as well as Sep15 and Usp18, which regulates Stat-1 and Irf7 [38], in NCI-60 cell lines provided further support that Sep15 may interfere with Stat-mediated inflammation and apoptosis, and this will have to be investigated further.

Other studies have shown that ablation of TR1, an important regulator of redox homeostasis protecting cells against oxidative stress [15,39,40], inhibited cell growth and apoptosis [17,41,42]. In absence of TR1 in liver and other tissues, activation of the transcription factor Nrf2 and subsequent up-regulation of enzymes of the glutathione system are expected as part of an cell survival strategy [9,41,43]. Unexpectedly, neither Nrf2-regulated genes, nor glutathione-synthesizing or –reducing enzymes were significantly affected in our shTR1 or shTR1/Sep15 colon cancer cells. This lack of Nrf2-increase may highlight the importance of context, as this is one of the few studies to investigate gene changes in the absence of TR1 specifically in colon cancer cells. Our results further showed that cells lacking TR1 responded with an increase in Usp18 mRNA expression, but without concomitant increases in the Stat-1-regulated genes as was seen in shSep15 cells. Usp18 inhibits Stat-signaling under physiological conditions in pancreatic beta-cells [38], but its role in colorectal cancer remains unknown. Thus, it is unclear if the high levels of Usp18 mRNA in shSep15 and shTR1 colon cancer cells support our results.

Combined down-regulation of Sep15 and TR1 resulted in unexpected recovery of the malignant phenotype in cancer cells, suggesting a compensatory or inhibitory mechanism between Sep15 and TR1, possibly because of changes in the regulation of the Apc-Wnt/β-catenin pathway. Inactivation of the tumor suppressor Apc (S3B Fig) is associated with activation of Wnt/β-catenin (S3C Fig), and is usually a key initiating event for tumorigenesis in familial and sporadic colon cancers [44]. This fits with our phenotypic observations in shTR1/shSep15 cells, and our analyses point to a significant involvement of the downstream effectors of the Wnt/β-catenin signaling pathway. For example, proliferin-1 (*Prl2c2*), the second highest up-regulated gene compared to controls, positively regulates angiogenesis and cell proliferation [45], and activates the Wnt pathway [46]. Furthermore, Tenascin (*Tnc*), the highest up-regulated gene in shTR1/shSep15 cells compared to controls, is found in restricted locations in normal adult tissues, including colon mucosa [47], and is a predictor of poor prognosis in colorectal cancer [48]. Increased expression of tenascin may facilitate tumor cell invasion and metastasis [47,48], possibly explaining maintenance of the cancer phenotype in shTR1/shSep15 cells. It is difficult to deduce from this type of analysis whether a direct connection exists between Wnt/β-catenin signaling and either Sep15 or TR1, or whether the Wnt/β-catenin pathway is only induced after down-regulation of both Sep15 and TR1. However, the changes in this pathway appear to be logical for colon tumorigenesis and may explain the loss of anti-cancer effects in double-knockdown cells. Kipp *et al*. reported that oxidative activation of the Wnt-signaling pathway may occur through selenium restriction [49]. In regard to selenium status in relation to the data reported herein, our previously published results showed that dietary selenium status had no additional effects on the ability of shSep15 colon cancer cells to form lung metastases [18]. These data, however, are too limited to speculate on the possible effects of selenium status on the observations with Sep15 and TR1 single knockdowns, or the double knockdown, and such assessments must await future studies.

In conclusion, we demonstrated that transfection of mouse colon carcinoma CT26 cells with either shSep15 or shTR1 partially reversed the cancer phenotype. This response was mediated by two different pathways. Targeted down-regulation of Sep15 resulted in up-regulation of Stat-mediated inflammatory pathways important in prevention of tumor promotion and maintenance. Consequently, our results provide evidence that Sep15 may be involved in the regulation of not only cancer initiation [7], but also tumor promotion. Dual tumor development functions have already been described for TR1 [8,17,31]. Reduced proliferation and decreased formation of lung metastases in shTR1 cells support a role for TR1 in the maintenance of colon tumors consistent with previous observations in lung cancer cells [17]. However, the molecular pathways through which these two selenoproteins with redox-functions accomplish these goals appear to be very different, because combined down-regulation of TR1 and Sep15 no longer resulted in the anti-cancer changes observed upon individual knockdown of either TR1 or Sep15. Instead, components of the Wnt/β-catenin pathway were significantly up-regulated, presumably to compensate for loss of two antioxidant systems, resulting in recovery of the original cancer phenotype. Based on this and previous observations, both Sep15 and TR1 appear to be involved in tumorigenesis, either at the stages of initiation and/or promotion [7,18,19,50], and thus may be potential targets for cancer therapy. Since the differential expression of these two selenoproteins has been linked to variable cancer incidence and mortality, they also may serve as viable biomarkers for identifying individuals who are more likely to respond to dietary interventions.

## Supporting Information

S1 Fig mRNA levels of selenoproteins. mRNA levels of **(A)** GPx1; **(B)** GPx2; and **(C)** SelM in cells stably transfected with the control, shSep15, shTR1 or shTR1/shSep15 constructs, as measured using real-time RT-PCR, and expressed relative to *Gapdh*. Columns, mean (*n* = 3–6); bars, SE; (*P<0.05).
(TIF)

S2 Fig Protein expression of Afp and β-catenin. Protein expression of (**A**) Afp, and (**B**) phosphorylated-β-catenin, in cells stably transfected with the control, shSep15, shTR1 or shTR1/shSep15 constructs, as determined by Western blotting, and expressed relative to α-tubulin.
(TIF)

S3 Fig Ingenuity Pathway Analyses. **(A)** Network analysis of shSep15 vs. control cells with *Stat-1* as the central molecule. Network of genes significantly changed exclusively in shTR1/shSep15 cells compared to plasmid-transfected controls showed involvement of regulators in the Wnt/β-catenin pathway, including **(B)** *Apc*, and **(C)** *Wnt*, *Ctnnbip1*, *Tnc* and *Axin1*.
(TIF)

S1 Table Real-time quantitative RT-PCR primers. List of primers used to measure the mRNA levels of selenoproteins and genes regulated by targeted down-regulation of Sep15, TR1 or both, by real-time quantitative RT-PCR.
(DOCX)

S2 Table Microarray summary of the 20 most strongly up-/down-regulated gene signals among statistically significant gene changes compared to controls (*P<*0.05). (DOCX)

## References

[1] American Cancer Society. Cancer Facts & Figures 2013. Atlanta: American Cancer Society, 2013.

[2] Duffield-Lillico AJ, Dalkin BL, Reid ME, Turnbull BW, Slate EH, Jacobs ET, et al Selenium supplementation, baseline plasma selenium status and incidence of prostate cancer: an analysis of the complete treatment period of the Nutritional Prevention of Cancer Trial. BJU Int. 2003;91: 608–612. 12699469 10.1046/j.1464-410x.2003.04167.x

[3] Jao SW, Shen KL, Lee WP, Ho YS. Effect of selenium on 1,2-dimethylhydrazine-induced intestinal cancer in rats. Dis Colon Rectum. 1996;39: 628–631. 8646947 10.1007/BF02056940

[4] Almondes KG, Leal GV, Cozzolino SM, Philippi ST, Rondó PH. The role of selenoproteins in cancer. Rev Assoc Med Bras. 2010;56: 484–488. 20835649 10.1590/s0104-42302010000400025

[5] Apostolou S, Klein JO, Mitsuuchi Y, Shetler JN, Poulikakos PL, Jhanwar SC, et al Growth inhibition and induction of apoptosis in mesothelioma cells by selenium and dependence on selenoprotein SEP15 genotype. Oncogene. 2004;23: 5032–5040. 15107826 10.1038/sj.onc.1207683

[6] Jablonska E, Gromadzinska J, Sobala W, Reszka E, Wasowicz W. Lung cancer risk associated with selenium status is modified in smoking individuals by Sep15 polymorphism. Eur J Cancer. 2008;47: 47–54. 10.1007/s00394-008-0696-9 18239845

[7] Tsuji PA, Carlson BA, Naranjo-Suarez S, Yoo MH, Xu X, Fomenko DE, et al Knockout of the 15 kDa selenoprotein protects against chemically-induced aberrant crypt formation in mice. PLoS One. 2012;7: e50574 10.1371/journal.pone.0050574 23226526 PMC3514276

[8] Yoo MH, Carlson BA, Tsuji PA, Tobe R, Naranjo-Suarez S, Lee BJ, et al Selenoproteins harboring a split personality in both preventing and promoting cancer In: Hatfield DL, Berry MJ, Gladyshev VN, editors. Selenium—Its molecular biology and role in human health. 3rd ed New York: Springer Science + Business Media, LLC; 2012.

[9] Brigelius-Flohé R, Müller M, Lippmann D, Kipp AP. The yin and yang of nrf2-regulated selenoproteins in carcinogenesis. Int J Cell Biol. 2012;2012: 486147 10.1155/2012/486147 22654914 PMC3357939

[10] Peters U, Chatterjee N, Hayes RB, Schoen RE, Wang Y, chanock SJ, et al Variation in the selenoenzyme genes and risk of advanced distal colorectal adenoma. Cancer Epidemiol Biomarkers Prev. 2008;17: 1144–1154. 10.1158/1055-9965.EPI-07-2947 18483336

[11] Carlson BA, Yoo MH, Tobe R, Mueller C, Naranjo-Suarez S, Hoffman VJ, et al Thioredoxin reductase 1 protects against chemically induced hepatocarcinogenesis via control of cellular redox homeostasis. Carcinogenesis. 2012;33: 1806–1813. 10.1093/carcin/bgs230 22791808 PMC3514905

[12] Kasaikina MV, Hatfield DL, Gladyshev VN. Understanding selenoprotein function and regulation through the use of rodent models. Biochim Biophys Acta. 2012;1823 1633–1642. 10.1016/j.bbamcr.2012.02.018 22440326 PMC3408893

[13] Labunskyy VM, Hatfield DL, Gladyshev VN. Selenoproteins: Molecular pathways and physiological roles. Physiological Reviews. 2014;94: 739–777. 10.1152/physrev.00039.2013 24987004 PMC4101630

[14] Arnér ES, Holmgren A. The thioredoxin system in cancer. Semin Cancer Biol. 2006;16: 420–426. 17092741 10.1016/j.semcancer.2006.10.009

[15] Yoo MH, Xu XM, Carlson BA, Patterson AD, Gladyshev VN, Hatfield DL. Targeting thioredoxin reductase 1 reduction in cancer cells inhibits self-sufficient growth and DNA replication. PLOS One. 2007;2: e1112 17971875 10.1371/journal.pone.0001112 PMC2040202

[16] Korotkov KV, Kumaraswamy E, Zhou Y, Hatfield DL, Gladyshev VN. Association between the 15-kDa selenoprotein and UDP-glucose:glycoprotein glucosyltransferase in the endoplasmic reticulum of mammalian cells. J Biol Chem. 2001;276: 15330–15336. 11278576 10.1074/jbc.M009861200

[17] Yoo MH, Xu XM, Carlson BA, Gladyshev VN, Hatfield DL. Thioredoxin reductase 1 deficiency reverses tumor phenotypes and tumorigenicity of lung carcinoma cells. J Biol Chem 2006;281: 13005–13008. 16565519 10.1074/jbc.C600012200

[18] Irons R, Tsuji PA, Carlson BA, Ouyang P, Yoo MH, Xu XM, et al Deficiency in the 15 kDa selenoprotein inhibits tumorigenicity and metastasis of colon cancer cells. Cancer Prev Res. 2010;3: 630–639. 10.1158/1940-6207.CAPR-10-0003 20388823 PMC2865577

[19] Tsuji PA, Naranjo-Suarez S, Carlson BA, Tobe R, Yoo MH, Davis CD. Deficiency in the 15 kDa selenoprotein inhibits colon cancer cell growth. Nutrients. 2012;3: 805–817. 10.3390/nu3090805 PMC3257736 22254125

[20] Kumaraswamy E, Korotkov KV, Diamond AM, Gladyshev VN, Hatfield DL. Genetic and functional analysis of mammalian Sep15 selenoprotein. Methods Enzymol. 2002;347: 187–197. 11898406 10.1016/s0076-6879(02)47017-6

[21] Wright ME, Diamond AM. Polymorphisms in selenoprotein genes and cancer In: Hatfield DL, Berry MJ, Gladyshev VN, editors. Selenium—Its molecular biology and role in human health. 3rd ed New York: Springer Science + Business Media, LLC; 2012.

[22] Xu XM, Yoo MH, Carlson BA, Gladyshev VN, Hatfield DL. Simultaneous knockdown of the expression of two genes using multiple shRNAs and subsequent knock-in of their expression. Nat Protoc. 2009;4: 1338–1348. 10.1038/nprot.2009.145 19713955 PMC2753455

[23] Yoo MH, Carlson BA, Tsuji PA, Irons R, Gladyshev VN, Hatfield DL. Alteration of thioredoxin reductase 1 levels in elucidating cancer etiology. Methods Enzymol. 2010;474: 255–275. 10.1016/S0076-6879(10)74015-5 20609915 PMC3088101

[24] Holmgren A, M. B. Thioredoxin and thioredoxin reductase. Methods Enzymol. 1995;252: 199–208. 7476354 10.1016/0076-6879(95)52023-6

[25] Reinhold WC, Sunshine M, Liu H, Varma S, Kohn KW, Morris J, et al CellMiner: A Web-Based Suite of Genomic and Pharmacologic Tools to Explore Transcript and Drug Patterns in the NCI-60 Cell Line Set. Cancer Res. 2012;72: 3499–3511. 10.1158/0008-5472.CAN-12-1370 22802077 PMC3399763

[26] Hsieh CM, Yoshizumi M, Endege WO, Kho CJ, Jain MK, Kashiki S, et al APEG-1, a novel gene preferentially expressed in aortic smooth muscle cells, is down-regulated by vascular injury. J Biol Chem. 1996;271: 17354–17359. 8663449 10.1074/jbc.271.29.17354

[27] Hatfield DL, Tsuji PA, Carlson BA, Gladyshev VN. Selenium and selenocysteine: roles in cancer, health, and development. Trends in Biochemical Sciences. 2014;39: 112–120. 10.1016/j.tibs.2013.12.007 24485058 PMC3943681

[28] Clark LC, Combs GFJ, Turnbull BW, Slate E, Chalker D, Chow J, et al Effects of selenium supplementation for cancer prevention in patients with carcinoma of the skin. A randomized controlled trial. Nutritional Prevention of Cancer Study Group. JAMA. 1996;276: 1957–1963. 8971064

[29] Duffield-Lillico AJ, Reid ME, Turnbull BW, Combs GF, Slate EH, Fischbach LA, et al Baseline characteristics and the effect of selenium supplementation on cancer incidence in a randomized clinical trial: a summary report of the Nutritional Prevention of Cancer Trial. Cancer Epidemiol Biomarkers Prev. 2002;11: 630–639. 12101110

[30] Lippman SM, Klein EA, Goodman PJ, Lucia MS, Thompson IM, Ford LG, et al Effect of selenium and vitamin E on risk of prostate cancer and other cancers: the Selenium and Vitamin E Cancer Prevention Trial (SELECT). JAMA. 2009: 1.

[31] Irons R, Carlson BA, Hatfield DL, Davis CD. Both selenoproteins and low molecular weight selenocompounds reduce colon cancer risk in mice with genetically impaired selenoprotein expression. J Nutr 2006;135: 1311–1317. 10.1093/jn/136.5.1311 16614422

[32] Kasaikina MV, Fomenko DE, Labunskyy VM, Lachke SA, Qiu W, Moncaster JA, et al Roles of the 15-kDa selenoprotein (Sep15) in redox homeostasis and cataract development revealed by the analysis of Sep 15 knockout mice. J Biol Chem. 2011;286: 33203–33212. 10.1074/jbc.M111.259218 21768092 PMC3190948

[33] Villanueva A, Alsinet C, Yanger K, Hoshida Y, Zong Y, Toffanin S, et al Notch signaling is activated in human hepatocellular carcinoma and induces tumor formation in mice. Gastroenterology. 2012;143: 1660–1669. 10.1053/j.gastro.2012.09.002 22974708 PMC3505826

[34] Collier J, Sherman M. Screening for hepatocellular carcinoma. Hepatology. 1998;27: 273–278. 9425947 10.1002/hep.510270140

[35] Pensa S, Regis G, Boselli D, Novelli F, Poli V. STAT1 and STAT3 in tumorigenesis: two sides of the same coin? In: Stephanou A, editor. JAK-STAT Pathway in Disease. Austin, TX: Landis Bioscience; 2009.

[36] Lee SC, Chan J, Clement MV, Pervaiz S. Functional proteomics of resveratrol-induced colon cancer cell apoptosis: caspase-6-mediated cleavage of lamin A is a major signaling loop. Proteomics. 2006;6: 2386–2394. 16518869 10.1002/pmic.200500366

[37] Chan JY, Phoo MS, Clement MV, Pervaiz S, Lee SC. Resveratrol displays converse dose-related effects on 5-fluorouracil-evoked colon cancer cell apoptosis: the roles of caspase-6 and p53. Cancer Biol Ther. 2008;7: 1308–1312. 10.4161/cbt.7.8.6302 18497562

[38] Santin I, Moore F, Grieco FA, Marchetti P, Brancolini C, Eizirik DL. USP18 is a key regulator of the interferon-drivengene network modulating pancreatic beta-cell inflammation and apoptosis. Cell Death and Disease. 2012;3: e419 10.1038/cddis.2012.158 23152055 PMC3542594

[39] Ma X, Hu J, Lindner DJ, Kalvakolanu DV. Mutational analysis of human thioredoxin reductase 1. Effects on p53-mediated gene expression and interferon and retinoic acid-induced cell death. J Biol Chem. 2002;277: 22460–22468. 11953436 10.1074/jbc.M202286200

[40] Moos PJ, Edes K, Cassidy P, Massuda E, Fitzpatrick FA. Electrophilic prostaglandins and lipid aldehydes repress redox-sensitive transcription factors p53 and hypoxia-inducible factor by impairing the selenoprotein thioredoxin reductase. J Biol Chem. 2003;278: 745–750. 12424231 10.1074/jbc.M211134200

[41] Patterson AD, Carlson BA, Li F, Bonzo JA, Yoo MH, Krausz KW, et al Disruption of thioredoxin reductase 1 protects mice from acute Acetaminophen-induced hepatotoxicity through enhanced NRF2 activity. Chem Res Toxicol. 2013;26: 1088–1096. 10.1021/tx4001013 23697945 PMC6334300

[42] Gasdaska JR, Berggren M, Powis G. Cell growth stimulation by the redox protein thioredoxin occurs by a novel helper mechanism. Cell Growth Differ. 1995;6: 1643–1650. 9019170

[43] Suvorova ES, Lucas O, Weisend CM, Rollins MF, Merrill GF, Capecchi MR, et al Cytoprotective Nrf2 pathway is induced in chronically txnrd 1-deficient hepatocytes. PLoS One. 2009;4: e6158 10.1371/journal.pone.0006158 19584930 PMC2703566

[44] Fodde R. The APC gene in colorectal cancer. Eur J Cancer Prev. 2002;38: 867–871. 10.1016/s0959-8049(02)00040-0 11978510

[45] Wang JW, Jiang YN, Huang CY, Huang PY, Huang MC, Cheng WT, et al Proliferin enhances microvilli formation and cell growth of neuroblastoma cells. Neurosci Res. 2006 56: 80–90. 16876275 10.1016/j.neures.2006.05.011

[46] Wang XY, Yin Y, Yuan H, Sakamaki T, Okano H, Glazer RI. Musashi1 modulates mammary progenitor cell expansion through proliferin-mediated activation of the Wnt and Notch pathways. Mol Cell Biol. 2008 28: 3589–3599. 10.1128/MCB.00040-08 18362162 PMC2423292

[47] Juuti A, Nordling S, Louhimo J, Lundin J, Haglund C. Tenascin C expression is upregulated in pancreatic cancer and correlates with differentiation. J Clin Pathol. 2004;57: 1151–1155. 15509674 10.1136/jcp.2003.015818 PMC1770485

[48] Kressner U, Lindmark G, Tomasini-Johansson B, Bergström R, Gerdin B, Påhlman L, et al Stromal tenascin distribution as a prognostic marker in colorectal cancer. Br J Cancer. 1997;76: 526–530. 9275031 10.1038/bjc.1997.419 PMC2227978

[49] Kipp A, Banning A, van Schothorst EM, Méplan C, Schomburg L, Evelo C, et al Four selenoproteins, protein synthesis, and Wnt signaling are particularly sensitive to limited selenium intake in mouse colon. Mol Nutr Food Res. 2009;53: 1561–1572. 10.1002/mnfr.200900105 19810021

[50] Hatfield DL, Gladyshev VN. The Outcome of Selenium and Vitamin E Cancer Prevention Trial (SELECT) reveals the need for better understanding of selenium biology. Mol Interv. 2009;9: 18–21. 10.1124/mi.9.1.6 19299660 PMC2718722

