# Supplementary figures and images for: The 15kDa Selenoprotein and Thioredoxin Reductase 1 Promote Colon Cancer by Different Pathways
Source: PLoS One. 2015 Apr 17;10(4):e0124487. doi: 10.1371/journal.pone.0124487 (PMC4401539; doi:10.1371/journal.pone.0124487)

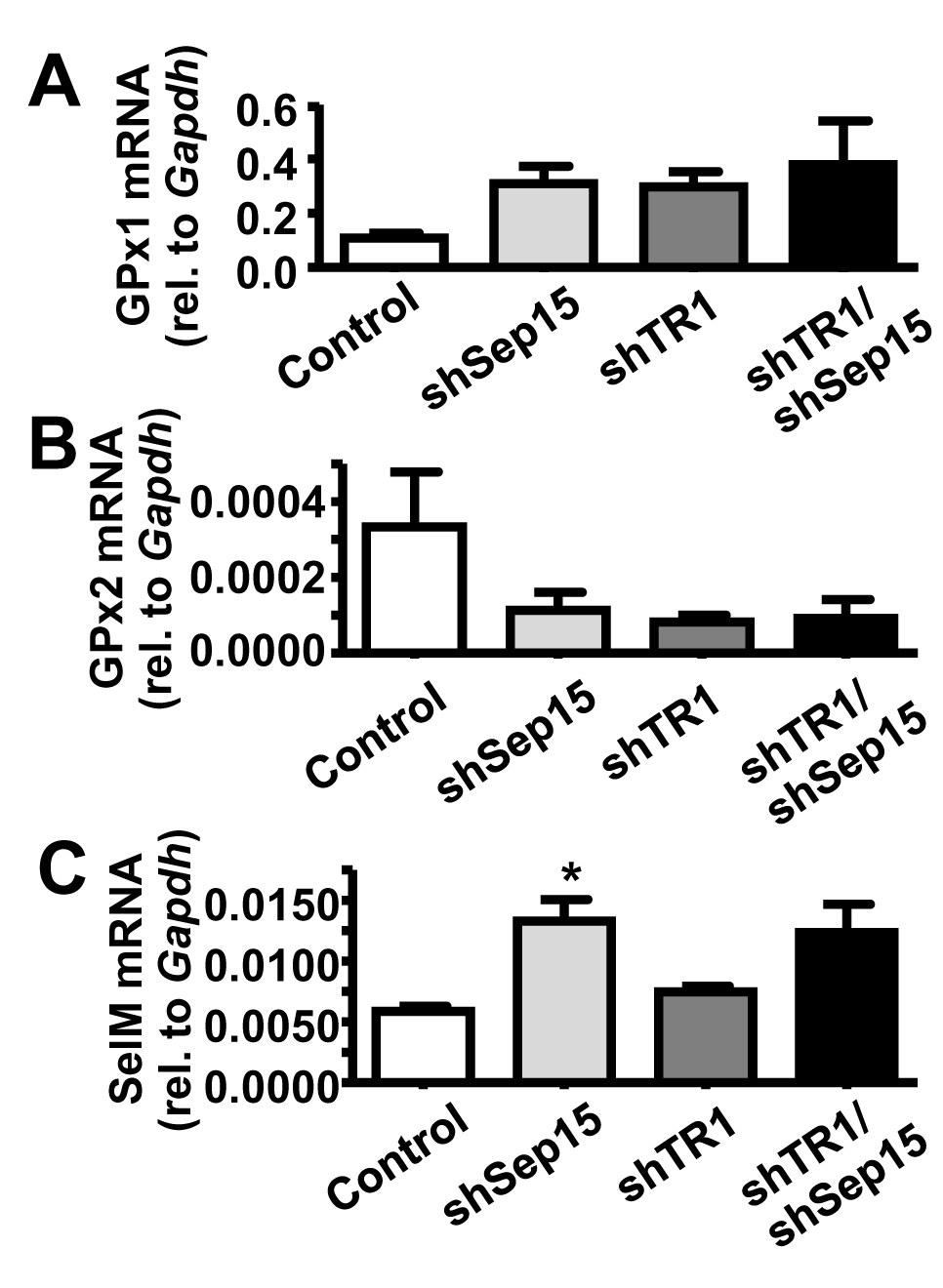

Supplement: S1 Fig — mRNA levels of (A) GPx1; (B) GPx2; and (C) SelM in cells stably transfected with the control, shSep15, shTR1 or shTR1/shSep15 constructs, as measured using real-time RT-PCR, and expressed relative to Gapdh. Columns, mean (n = 3–6); bars, SE; (*P<0.05). (TIF) [file pone.0124487.s001.tif]

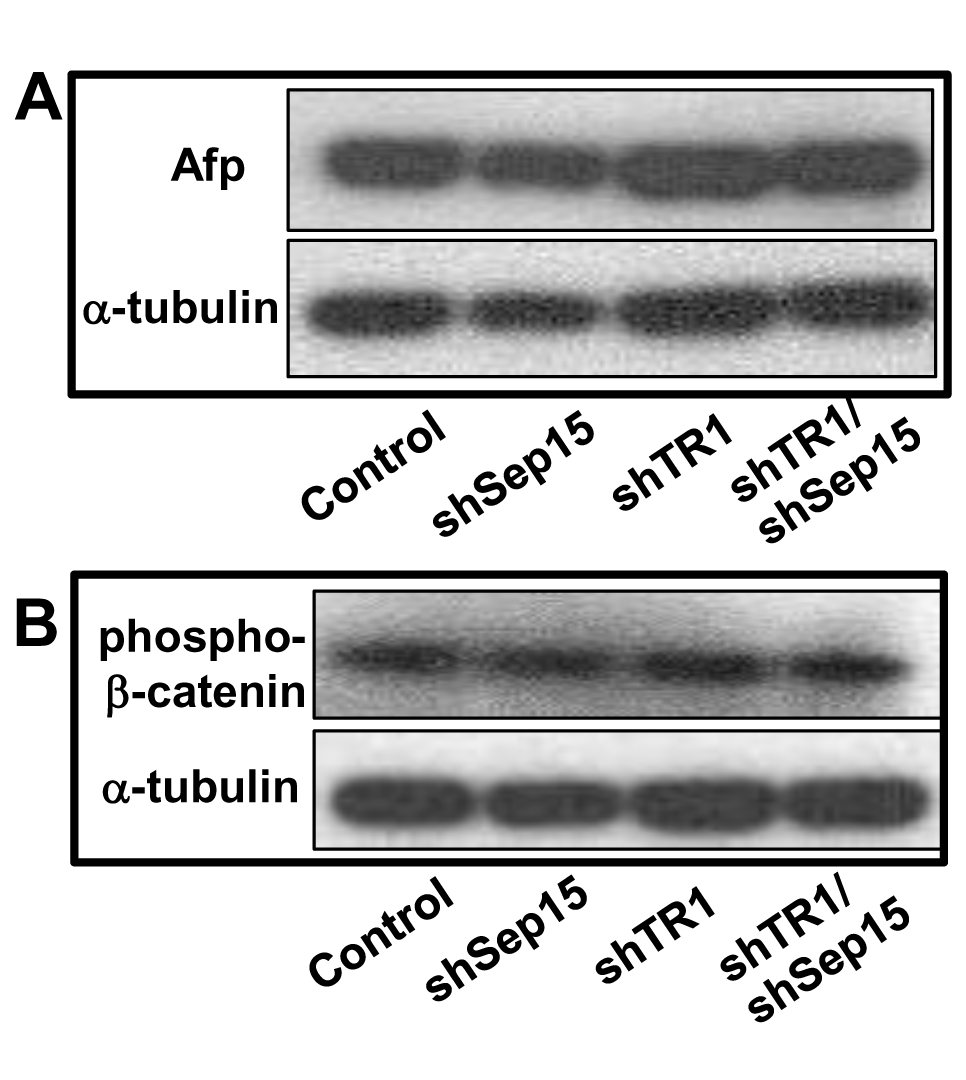

Supplement: S2 Fig — Protein expression of (A) Afp, and (B) phosphorylated-β-catenin, in cells stably transfected with the control, shSep15, shTR1 or shTR1/shSep15 constructs, as determined by Western blotting, and expressed relative to α-tubulin. (TIF) [file pone.0124487.s002.tif]

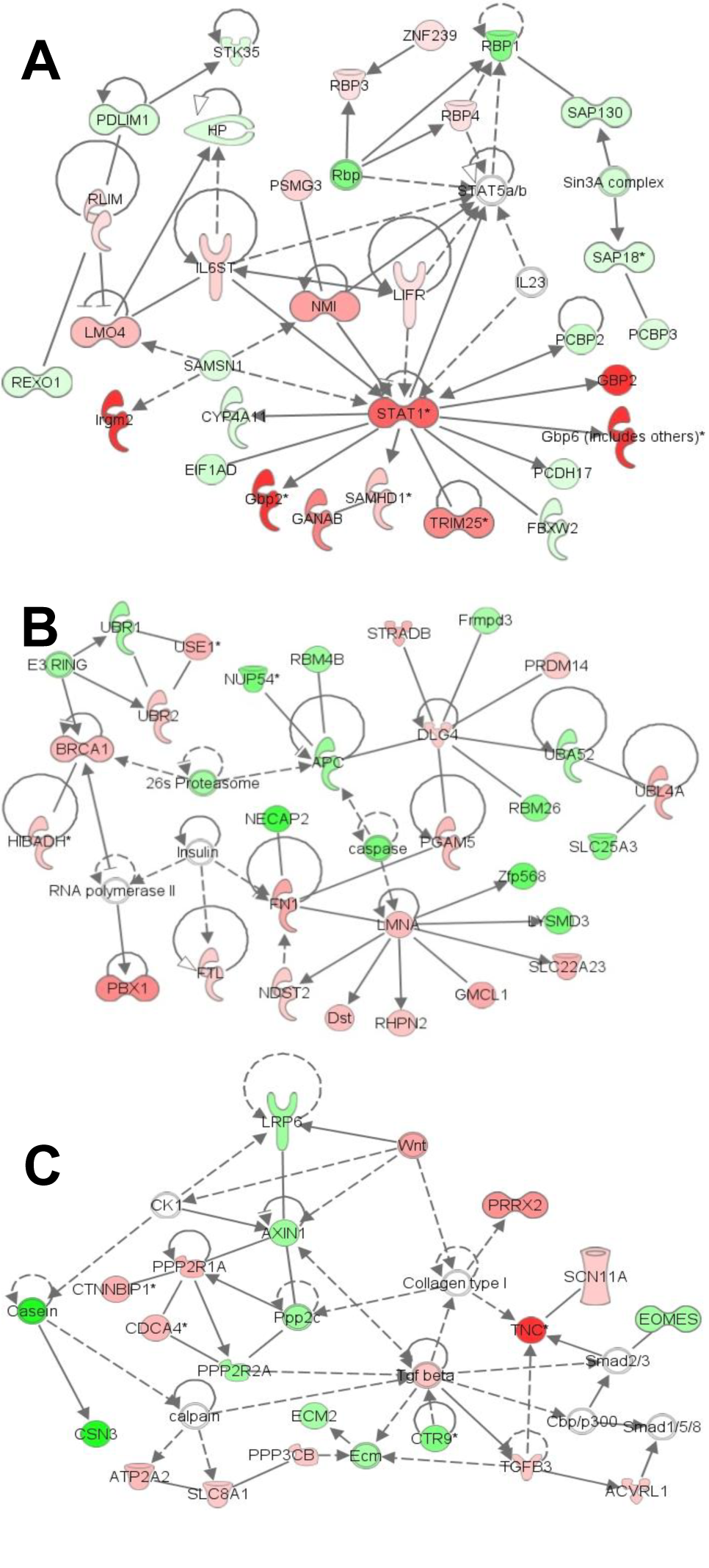

Supplement: S3 Fig — (A) Network analysis of shSep15 vs. control cells with Stat-1 as the central molecule. Network of genes significantly changed exclusively in shTR1/shSep15 cells compared to plasmid-transfected controls showed involvement of regulators in the Wnt/β-catenin pathway, including (B) Apc, and (C) Wnt, Ctnnbip1, Tnc and Axin1. (TIF) [file pone.0124487.s003.tif]
