# Supplementary material for: The 15kDa Selenoprotein and Thioredoxin Reductase 1 Promote Colon Cancer by Different Pathways
Source: PLoS One. 2015 Apr 17;10(4):e0124487. doi: 10.1371/journal.pone.0124487 (PMC4401539; doi:10.1371/journal.pone.0124487)
Supplement: S1 Table — List of primers used to measure the mRNA levels of selenoproteins and genes regulated by targeted down-regulation of Sep15, TR1 or both, by real-time quantitative RT-PCR. (DOCX) [file pone.0124487.s004.docx]

**Table S1**.

| **Target gene** | **Accession No** | **Sequence** |
| --- | --- | --- |
| Afp | NM_007423.4 | fwd GGGAATGGCCGACATTTTCAT  rev GCAGTGGCTGATACCAGAGT |
| Apc | NM_007462.3 | fwd TGGAGAAGCGTGCACAGCGAA  rev GCCGGCCAGCTTCATGGGAG |
| Casp6 | NM_009811.3 | fwd GAAGTGTTCGATCCAGCCGA  rev CTTGAAGTCGACACCTCGTGA |
| Casp12 | NM_009808.4 | fwd AGGGGAAAGTGCGAGTTTCAT  rev CTGTCTCCATTTCCTGAGCTGT |
| Ccnb1 | NM_172301.3 | fwd CGGAGAGGTTGACGTCGAGCAG  rev AGCGCTAAGCAGAAAGCCCCA |
| Ccnb1ip1 | NM_001111119.1 | fwd AGCAGCAAGTCTGCAGCAGG  rev ACCAAACAGGAAGCCACACGCC |
| Ccr1 | NM_009912.4 | fwd CTGGAAACACAGACTCACTGTC  rev GCCCACCACTCCAATGATGA |
| Gapdh | NM_008084 | fwd ATGTGTCCGTCGTGGATCT  rev GTTGAAGTCGCAGGAGACAA |
| Gbp-1 | NM_010259 | fwd GATTTCTCCCTGGATCTGGA  rev CACAGGCGAGGCATATTAAA |
| Gbp-6 | NM_194336.2 | fwd CTCTGGGACCAGGTTGCTAT  rev TGGATGCTTGCATTCTGGGT |
| Gpx1 | NM_008160 | fwd CAGGAGAATGGCAAGAATGA  rev GAAGGTAAAGAGCGGGTGAG |
| Gpx2 | NM_030677 | fwd ATCAAACGGCTCCTCAAAGT  rev GGGACGATATTCAGGGAATG |
| Ifi44 | NM_133871.2 | fwd CGGCTTGCACACAGATGATG  rev TGGTTTCATGGAATCGAACTGG |
| Irf7 | NM_016850.3 | fwd AGCTTGGATCTACTGTGGGC  rev GGGTTCCTCGTAAACACGGT |
| Ifnγ | NM_008337 | fwd GGCATAGATGTGGAAGAAAA  rev GGCCTGATTGTCTTTCAA |
| Il2rg | NM_013563.3 | fwd TGTTGGTTGGAACGAATGCC  rev AGGCCGAAAAGTTCCCTTGG |
| Nre2l2 | NM_010902.3 | fwd GGTTGCCCACATTCCCAAAC  rev GCAAGCGACTCATGGTCATC |
| Sel M | NM_053267 | fwd GATTGGAACCGTCTTCGAG  rev GTGCTTCATCACCAGGTTGT |
| Sep15 | NM_053102 | fwd TGGAACACAGACAGTGTGGA  rev TGACCAATGTAAGCATGCAA |
| Speg | NM_001085370.1 | fwd GAGACGAGAGCGAGCATTC  rev GAGTTCGAGGTCCGAGGTTG |
| Stat-1 | NM_001205313.1 | fwd GGATCGCTTGCCCAACTCTT  rev TTCCCTCCTGGGCCTGATTA |
| Tnc | NM_011607.3 | fwd CGACCTCACACACGAAGACA  rev CACTTCTTCCGTGGATGCCT |
| Tr1 | NM_015762 | fwd CTACAGACCATTGCCTTGCT  rev ACCTCCTACCCACAAGATCC |
| Usp18 | NM_011909.2 | fwd CAACGTGCCGTTGTTTGTCC  rev CAGCCGCTCTGTCAAGTCTG |
