# Supplementary material for: The 15kDa Selenoprotein and Thioredoxin Reductase 1 Promote Colon Cancer by Different Pathways
Source: PLoS One. 2015 Apr 17;10(4):e0124487. doi: 10.1371/journal.pone.0124487 (PMC4401539; doi:10.1371/journal.pone.0124487)
Supplement: S2 Table — (DOCX) [file pone.0124487.s005.docx]

**Table S2.**

| **Most up-regulated gene signals** | **shSep15 - Control** | |  | **shTR1 - Control** | |  | **shTR1/shSep15 - Control** | |
| --- | --- | --- | --- | --- | --- | --- | --- | --- |
|  | **Fold change** | **Gene** |  | **Fold change** | **Gene** |  | **Fold change** | **Gene** |
|  | 136.4 | Ifi44 |  | 9.0 | Ccr1 |  | 9.0 | Tnc |
|  | 65.1 | Usp18 |  | 6.7 | Gm2666 |  | 7.5 | Prl2c2 |
|  | 31.1 | Gbp2 |  | 6.3 | Cd68 |  | 7.4 | Slc14a1 |
|  | 30.1 | Gbp6 |  | 5.9 | Al607873 |  | 5.4 | Btc |
|  | 20.7 | Irgm2 |  | 5.8 | Speg |  | 5.1 | Ccr1 |
|  | 14.3 | Gbp1 |  | 5.2 | Dmxl2 |  | 4.7 | Ifitm1 |
|  | 14.3 | Xaf1 |  | 5.1 | Prkar1b |  | 4.5 | Pthlh |
|  | 12.4 | Phf11 |  | 3.9 | Gcnt2 |  | 4.2 | Ngef |
|  | 11.0 | Eif4g3 |  | 3.7 | Irs1 |  | 4.2 | Thbs2 |
|  | 9.9 | Fpgt |  | 3.6 | Lyst |  | 4.1 | Prrx1 |
|  | 9.9 | Irf7 |  | 3.5 | Abhd6 |  | 4.0 | Cldn6 |
|  | 9.4 | Atp8a1 |  | 3.4 | Mipol1 |  | 3.9 | Pla2g7 |
|  | 8.8 | Igrp |  | 3.4 | Cldn6 |  | 3.7 | Syt7 |
|  | 8.6 | Gvin1 |  | 3.3 | Rpp40 |  | 3.6 | Slc14a1 |
|  | 8.4 | Tmem47 |  | 3.3 | Lmo7 |  | 3.6 | Cpn1 |
|  | 8.4 | Bank1 |  | 3.2 | Ngef |  | 3.4 | Rgl1 |
|  | 7.5 | Spint2 |  | 3.1 | Pgap1 |  | 3.4 | Rasa3 |
|  | 7.2 | Parp14 |  | 3.1 | Ap1s3 |  | 3.4 | Mmd |
|  | 7.2 | Sfmbt2 |  | 3.0 | Tecpr1 |  | 3.3 | Limk1 |
|  | 6.8 | Tmem47 |  | 3.0 | Selp |  | 3.3 | Thbs2 |

| **Most down-regulated gene signals** | **shSep15 - Control** | |  | **shTR1 - Control** | |  | **shTR1/shSep15 - Control** | |
| --- | --- | --- | --- | --- | --- | --- | --- | --- |
|  | **Fold change** | **Gene** |  | **Fold change** | **Gene** |  | **Fold change** | **Gene** |
|  | 69.2 | Afp |  | 26.0 | Arhgef5 |  | 24.1 | Il2rg |
|  | 27.4 | Il2rg |  | 24.9 | Dppa2 |  | 18.5 | Dppa2 |
|  | 27.3 | Nefl |  | 21.0 | Il2rg |  | 6.4 | Nhedc1 |
|  | 23.9 | Klf2 |  | 10.4 | Lrrn4cl |  | 4.8 | Trim30 |
|  | 23.7 | Arhgef5 |  | 5.2 | Klf2 |  | 4.8 | Cyr61 |
|  | 21.2 | Dppa2 |  | 4.9 | Clu |  | 4.5 | Malat1 |
|  | 16.0 | Igfbp4 |  | 3.6 | Phrf1 |  | 4.4 | Cyr61 |
|  | 15.4 | Dppa2 |  | 3.6 | Tshz1 |  | 4.2 | Tshz1 |
|  | 13.3 | Lrrn4cl |  | 3.5 | Mfap5 |  | 4.1 | Prkx |
|  | 13.2 | Serpinf1 |  | 3.3 | Zfp191 |  | 4.0 | Zfp397 |
|  | 12.4 | Nefm |  | 3.2 | L2hgdh |  | 3.9 | Klf2 |
|  | 10.6 | Dppa4 |  | 3.1 | Zfp397 |  | 3.4 | Zadh2 |
|  | 10.6 | Il2rg |  | 2.8 | Atp1a3 |  | 3.2 | Gm13242 |
|  | 10.3 | Igfbp4 |  | 2.5 | Dennd2d |  | 3.0 | Csn3 |
|  | 9.9 | Ifitm1 |  | 2.4 | Anxa8 |  | 3.0 | Bptf |
|  | 9.7 | Krt20 |  | 2.4 | Ccdc80 |  | 3.0 | Kitl |
|  | 9.1 | Rhob |  | 2.4 | Jakmip3 |  | 2.9 | Taf15 |
|  | 8.3 | Col3a1 |  | 2.3 | Tmem119 |  | 2.9 | Zpf191 |
|  | 6. 7 | Tshz1 |  | 2.3 | Pcdhb16 |  | 2.9 | Tlk1 |
|  | 6.4 | Krt18 |  | 2.3 | Gldc |  | 2.9 | Itsn2 |
